# HIV-2 and its role in conglutinated approach towards Acquired Immunodeficiency Syndrome (AIDS) Vaccine Development

**DOI:** 10.1186/2193-1801-2-7

**Published:** 2013-01-11

**Authors:** Batul Diwan, Rupali Saxena, Archana Tiwari

**Affiliations:** School of Biotechnology, Rajiv Gandhi Proudyogiki Vishwavidyalaya, Bhopal, MP India

**Keywords:** Human Immunodeficiency Virus-2, Vaccine Development, Bioinformatics, Vaccinomics

## Abstract

Acquired Immunodeficiency Syndrome (AIDS) is one of the most critically acclaimed endemic diseases, caused by two lentiviruses HIV-1 and 2. HIV-2 displays intimate serological and antigenic resemblance to Simian Immunodeficiency Virus (SIV) along with less pathogenicity, lower infectivity and appreciable cross reactivity with HIV-1 antigens. The present era is confronted with the challenge to fabricate a vaccine effective against all clades of both the species of HIV. But vaccine development against HIV-1 has proven highly intricate, moreover the laborious and deficient conventional approaches has slackened the pace regarding the development of new vaccines. These concerns may be tackled with the development of HIV-2 vaccine as a natural control of HIV-1 that has been found in ancestors of HIV-2 i.e. African monkeys, mangabeys and macaques. Thereby, suggesting the notion of cross protection among HIV-2 and HIV-1. Assistance of bioinformatics along with vaccinomics strategy can bring about a quantum leap in this direction for surpassing the bottleneck in conventional approaches. These specifics together can add to our conception that HIV-2 vaccine design by *in silico* strategy will surely be a constructive approach for HIV-1 targeting.

**Electronic supplementary material:**

The online version of this article (doi:10.1186/2193-1801-2-7) contains supplementary material, which is available to authorized users.

## Introduction

The first acknowledged case that marked the commencement of AIDS dates back to the late 19th century and ever since it has been regarded as the most devastating pandemic with no definite cure available till date. It is the final stage of an immunodeficiency disorder caused by a retrovirus called *Human Immunodeficiency Virus* (HIV) (Amadi et al. 2008). The origin of HIV, on the basis of intimate similarity is related to another kind of virus which infected monkeys, known as the Simian Immunodeficiency Virus (SIV) (Worobey et al. 2010).

AIDS is characterized by the selective targeting of the CD4+/CD8+ T cells by HIV which fatally impairs the immune system. The window period for this retrovirus may last from several weeks to few months altogether before the *seroconversion* (detection of earliest antibodies in blood serum raised against HIV) (Taylor 2003). However, a person is said to be diagnosed with AIDS, only when the CD4+T cell count drops below 200 cells/microlitre, time period for which is indefinite and may last from few months to years (Mandell et al. 2009). This makes the immune system highly vulnerable to opportunistic infections.

## Human Immunodeficiency Viruses 1& 2

There are two closely related human lentiviruses causing AIDS, referred to as HIV-1 and HIV-2 (Adam et al. 2007). Although HIV-1 is more common and prevalent species of HIV, HIV-2 is relatively new and was discovered in West Africa in 1986 (Clavel et al. 1986). It has evolved as a result of independent cross species transmission events from sooty mangabeys to humans (Clavel et al. 1986). It is found to be more widespread in West Africa, India and Europe infecting about 1 to 2 million individuals (de Silva et al. 2008). Even though there are few differences between HIV-1 and HIV-2 (Table 1), both are perilous. HIV-1 and 2 shares various biological and genetic properties such as genome structures, mechanism of trans-activation and manner of CD4+T cell depletion (Guyader et al. 1987; Levy 1993). The virulence properties of HIV-2 vary appreciably from HIV-1 and may range from comparative attenuation in certain individuals to elevated pathogenicity in others (Reeves & Doms 2002).

**Table 1:** **Comparison of HIV-1 and HIV-2 characteristics**

***CHARACTERISTICS***	***HIV-1***	***HIV-2***
***Infectivity***	High	Low
***Virulence***	High	Low
***Heterosexual Spread***	Higher	Lower
***Vertical Transmission***	20-25%	≤5%
***Genetic Diversity***	--	Lower
***Prevalence***	Global	West Africa
***Origin***	Common Chimpanzee	Sooty Mangabey
***Time to Aids***	≤10 Years	≥20 Years

Although both viruses lead to immunodeficiency but (Clavel et al. 1986) HIV-2 in contrast to HIV-1 exhibits lesser rates of sexual transmission, slower disease progression , longer clinical latency periods lower viral load in the asymptomatic stage (Sousa et al. 2002) and significantly lower vertical and horizontal transmission rates (Reeves & Doms 2002).

Strains of HIV-1 are classified as Group M, Group N, Group O and Group P. Out of these, only group M is broadly dispersed in the world whereas, HIV 2 has six subtypes (or clades) ranging from A to F and of these, subtype A is most prevalent (De Cock et al. 1993; Reeves & Doms 2002). Studies have revealed a relatively resistant response of HIV-2 infected individual towards consequent HIV-1 infection (De Cock et al. 1993; Reeves & Doms 2002). The similarities that exist between HIV-1 and HIV-2 can be exploited for the development of a prospective vaccine which can potentially target HIV-1. The underlying principle behind this approach is to formulate a vaccine based on the conserved genetic sequences that are present in HIV-2. As previously mentioned, HIV-2 infected individual provides a cross protection against HIV-1. So, it is thought that a vaccine developed against HIV-2 can also show the caliber of targeting HIV-1 (which is difficult to target otherwise due to its highly mutative nature). This can be achieved with the help of upcoming bioinformatic strategies which is dealt in Later Section. The main advantage of such an approach is that it saves a lot of time and energy that is indispensably called for any wet lab approach based on a similar concept.

## Genetics

Although HIV-1 and HIV-2/SIV groups of viruses might have genetically diverged from each other about 50 to 60 years ago (Chen et al. 1996) but they still show a genetic similarity of about 40–50 percent (Sourial et al. 2005). Primary, regulatory and accessory genes in HIV-2 are similar to that of HIV-1 with slight differences that are discussed below:

A. **Polymerase gene (pol):** Three proteins are encoded by *pol* of HIV-2: endonuclease/integrase (IN), protease (PR) and reverse transcriptase (RT), which also has RNaseH activity. Studies demonstrate that HIV-2 *pol* shows comparable error prone propensity as that of HIV-1 (Bakhanashvili & Hizi 1992) on the other hand, its RNaseH activity is reported to be lesser by almost 10-folds than HIV-1 enzyme (Hizi et al. 1991).
B. **Structural Genes (*gag*&*env*)**: Extremely conserved *gag* proteins are basically same for both HIV-1 and HIV-2 (Voss et al. 1992). A myristoylated 55-kDa precursor cleaved by the viral protease generates the capsid protein p24 (CA); the nucleocapsid protein p7 (NC); the matrix protein p17 (MA); p1; p2; and p6. HIV-2’s envelope glycoprotein precursor is somewhat smaller in contrast to HIV-1, hence at times it is designated with lower sizes (i.e. gp140–145, gp105–110 and gp32–40 respectively).
C. **Regulatory and Accessory Genes:** RNA processing or splicing is a vital control component apart from the long terminal repeat (LTR) regions at the ends of the viral genome containing crucial sequences for transcriptional activation and termination. As compared to HIV-1, HIV-2 has less responsive LTR to cellular activation signals and has dissimilar response elements (Popper et al. 2000). Also transcriptional activation requires additional factors. These differences along with lower CD4 affinity seen with HIV-2’s gp120 may account for the reason that makes HIV-2 infected individuals to have a lower viral load than those infected with HIV-1 (Sousa et al. 2002). Also HIV-2 shows enhanced sensitivity to neutralization due to its ability to infect host cell independent of CD4 interaction (Figure?1 & 2) (Reeves & Doms 2002).

**Figure 1:**
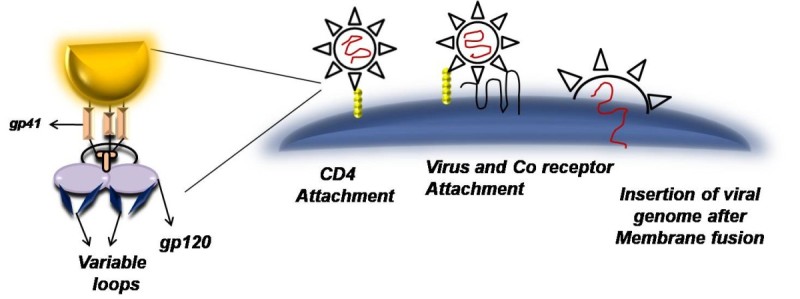
**CD4 Dependent infection:** Interaction between CD4 on T cell and viral gp120 leads to conformational changes in Env which further brings about interaction between co receptor and virus which further leads to fusion of viral membrane and insertion of viral genome in a host cell.

**Figure 2:**
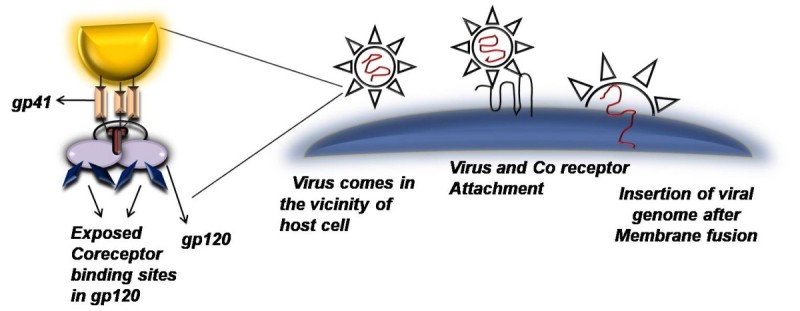
**CD4 Independent infection:** Interaction takes place directly between virus and co receptors to trigger membrane fusion and genome insertion process.

Studies also verify that among HIV-1 and HIV-2 infected individuals, T4 lymphocyte counts and T4:T8 ratios emerge to be declined less significantly in HIV-2 than in HIV-1 infected individuals (Kanki & Meloni 2004).

Genetic details of HIV-2 reveal many important factors responsible for slow progression and less virulent nature of HIV-2. Not only structural but also the regulatory genes demonstrate varied activity and its host attachment affinity is also lower compared to HIV-1 which might be the prime reason of HIV-2’s reduced virulent nature.

These data all together indicate that it is comparatively easier to target HIV-2 than HIV-1. Talking about immunity against HIV-2, humoral and T-cell immune response decline slowly compared to HIV-1 infected individual. So for vaccine design, immune response against HIV-2 can be studied which can offer hints regarding the way immune system reacts on its exposure.

## Immunity

HIV-2 illustrates attenuated infectivity which has constantly been a subject of curiosity among researchers for understanding its *immunopathogenesis*. Also, studies highlight the fact that the strong antibody response is generated by infected people against antigens encoded by *gag*, *pol*, and *env* (Chanock et al. 1987; Girard & Valette 1988). The broadness of neutralizing antibody reaction against HIV-2 is divergent from that of HIV-1 (Fenyo & Putkonen 1996). Cervicovaginal secretions of infected women demonstrated that one-third of them generated IgA response to HIV-2 envelope antigens confirming lesser viral replication as compared to HIV-1. HIV-2 infected individuals also show prominent cross-reactivity by IgG and IgA against heterologous envelope antigens in contrast to HIV-1 for this compartment (Belec et al. 1996). This might be the rationale behind dissimilar heterosexual transmission rates for one type of HIV in individuals infected by other type. This elucidation maintains the belief that HIV-2 infection protects against subsequent HIV-1 infection but not the vice versa. While some predicted the exact contrary detail that HIV-2 infection does not protect against consecutive HIV-1 infections (Greenberg 2001; Schim van der Loeff et al. 2001). In depth studies on HIV-1 & HIV-2 is required to conclude this debate and reveal several hidden facts. Nevertheless, these perceptions of cross protection among HIV-2 and HIV-1 can add to our comprehension whether viral variability can be conquered for the design of broadly reactive vaccine candidate against all subtypes (or clades).

## Necessity of Vaccine

Successful anti-HIV vaccine is an immediate need considering its pandemic in certain regions of the world including West Africa, China and Southeast Asia to halt this wave of spread. But its development has been a major challenge till date for scientists as HIV demolishes immune system intended to fight against it, also its genetic material remains in dormant form and thus escapes the immune system. It has quite a few subtypes each differing from one another and even in each subtypes it constantly mutates itself and lastly no satisfactory animal model exist for trials. However, non human primates may possibly appear as a significant option (Bernstein 2010). Even after the advancement in medication pattern new infections still persists and an effective vaccine is required to completely stop its spread. Till date more than 60 phase I, II and III trials are going on around the world but a truly effective vaccine is still far away from a stage where it can be said as achieved.

Hopes still persist for discovery of vaccine as many researches and findings elucidate production of broadly neutralizing antibodies targeting different types of HIV which can be the basis for new research in the vaccine development.

## Conventional Attempts

Numerous ongoing researches have taken into account various aspects for targeting HIV-2 each one of which can notably contribute towards its targeting.

Five major subtypes (or clades) of HIV-2 from A to E have already been reported to exist in certain human population (Gao et al. 1994). But a new highly divergent HIV-2 subtype (or clade) designated as F is the recent addition to the presently existing classes supporting the belief that new HIV-2 subtypes evolve from independent cross-species transmission of SIV_sm_ to the human population (Smith et al. 2008). Comparative analysis of SIV_sm_ and HIV-2 with HIV-1 in the macaques model demonstrated that pathogenic SIV_sm_ infection was neutralization resistant similar to HIV-1 infection in humans. On contrary, HIV-2 infected macaques remain susceptible to neutralization and exhibit cross reactivity on account of its steady biological properties (Laurén et al. 2006).

Vaccine trials carried out in macaque's model revealed that active immunization with iscoms (immune stimulating complex) using native inactivated viral proteins followed by iscom coupled V3 derived peptide booster doses induced protective immunity against HIV-2 infection in cynomologus monkeys (Putkonen et al. 1994). In another study, immunogenicity and neutralizing response of recombinant envelope proteins derived from the lone administration of reference primary HIV-2ALI (one kind of HIV-2 isolate) or in different prime-boost combinations in the mouse model was investigated. It was found that broadly reactive HIV-2 Nabs can be obtained by using a vaccinia virus vector-prime/rpC2-C3 (recombinant peptide containing C2 V3 C3 envelope region) boost immunization strategy which suggested a potential relationship between escape to neutralization (Marcelino et al. 2010). Similarly, the efficacy of a recombinant HIV-2 canarypox (ALVAC HIV-2 which uses canarypox virus which cannot harm human) vaccine given alone or in combination with HIV-2 envelope gp125 or HIV-2 V3 synthetic peptides was inspected in 14 cynomologus monkeys. Four out of ten monkeys immunized with ALVAC HIV−2 plus HIV-2 gp125 or V3 peptides were found to be protected (Andersson et al. 1996).

The aforementioned studies indicate the existence of certain conserved regions in HIV-2 against which significant immune response was seen in animal models. Several experiments were done which not only verified this, but also confirmed targeting capacity of some monoclonal antibodies specific to them. In a study done by Mannervik M *et al.,* synthetic peptides of external envelope glycoprotein gp125 based on HIV-2_SBL6669_ were used to recognize its continuous antigenic sites. It verified seroreactivity to few distinct linear sites on gp125 analogous to HIV-1 gp120 in case of molecular arrangement (Marcelino et al. 2006). In another investigation, neutralizing Ab’s targets for HIV-2 glycoprotein were mapped strictly considering the function of V3 region. Two discrete immunogenic sites were identified, first in the area with the conserved motif *Phe-His-Ser* (amino acid 315–317) second in the immediacy of the COOH-terminal cysteine *Trp-Cys-Arg* (amino acid 329–331). It was concluded that these two can possibly interact as a single discontinuous site representing the importance of V3 (Andersson 2001). These immunogenic sites in V3-loop were further characterized and the significance of different configurations of peptides corresponding to this region was studied and attained outcomes maintained the hypothesis that the well conserved motifs of the HIV-2_SBL6669_ V3 region i.e. FHSQ and WCR are crucial targets for neutralizing antibodies. These motifs can play a vital role for the designing of a potential HIV-2 vaccine (Dimonte et al. 2011; Morner et al. 1999).

On the other hand the role of humoral immunity in the evolution of the HIV-2 env C2V3C3 regions was examined in the context of their antibody response (IgG and IgA) against it. IgG antibody against C2V3C3 efficiently restricted viral population and escape while IgA successfully targeted C3 region (Borrego et al. 2008). Recently molecular, evolutionary and structural comparison of the these envelope regions from both HIV-1 and HIV-2 was done which suggested that in HIV-2 the V3 loop is much less exposed than C2 and C3 due to a physical interaction with both whereas its conserved nature is constant with lack of immunodominancy *in vivo* (Barroso et al. 2011).

The capacity of antibodies against such conserved domains was explored by generating them in rabbits against HIV-2 V2 and V3 regions to interact with glycosylated and deglycosylated protein, to hinder with gpl05-CD_4_ interface. It revealed that V2 and V3 regions are not directly involved in the gp105 binding site for the CD_4_ receptor (Babas et al. 1994). The production and characterization of MAbs (monoclonal antibodies) against these V3 and C3 regions of the gp120 of HIV-2ROD suggested that the V3 region of HIV-2 has a similar function to that of HIV-1 in the infection process differing from earlier result which indicated lack of immunodominancy of V3 and specified that V3 sequence of HIV-2 may be a useful target in an animal model for HIV vaccine development (Matsushita et al. 1995). In addition, the neutralization capacity of the purified anti HIV-2 V3 MAbs i.e. conformation-sensitive (3C4) and a linear site-specific (7C8) and their respective papain-generated Fab fragments suggested that whole 7C8 and 3C4 MAbs were sterically hindered from neutralizing HIV-2. Alternatively the smaller size of Fab fragments capably accessed the virion surface V3 region (Sourial & Nilsson 2008). Further proceeding in this direction, the crystal structure of the Fab fragment of 7C8 was presented for structural analysis which showed a deep and narrow highly hydrophobic antigen-binding cleft (Uchtenhagen et al. 2011).

Some distinctive details apart from the existence of conserved residues and generation of neutralizing antibodies against them were also revealed which might be valuable in some way or other. H. Akimoto *et al.*, found that the envelope glycoprotein of HIV-2 (not of HIV-1), could bind to CD4 and CD8 molecules on T cells α-chain (but not the P-chain) and induce phosphorylation of protein tyrosine kinase p56^1ck^(lymphocyte specific protein tyrosine kinase) in CD8+ T cells. Also production of β-chemokines in response to HIV-2 envelope glycoprotein was significantly higher as compared to the response to HIV-1 envelope glycoprotein which was related to signal transduction into CD8+ T cells and the resultant β-chemokines production in HIV-2 infection. This might explain the difference in virulence and disease manifestations between HIV-1 and HIV-2 infection (Cavaleiro et al. 2000).

Apart from envelope region *gag* was demonstrated to be most immunogenic specifically p26 region. The steady rapport between Gag specific immune responses and viral regulation indicated that, T cell responses are crucial in determining the better outcome of HIV-2 infection which may add to the understanding of relation between HIV-2 controlled infection and protective immunity for the design of HIV vaccine (Leligdowicz et al. 2007). Comparison of homologous and cross reactive Gag-specific T-cell responses between HIV-1 and HIV-2 infected patients for their gag peptides revealed that homologous Gag-specific T-cell responses were broader and stronger in HIV-2 infected patients as compared to HIV-1. In contrast cross reactive T-cell response was narrower and weaker in HIV-2 as compared to HIV-1 infected patients. This cross reactive response indicated HIV-1/HIV-2 Gag sequence similarities. Thus HIV-2-specific T-cell responses control HIV-2 replication restraining viral diversification (Jennes et al. 2008). The capsid region of Gag may be better processed and presented on MHC in contrast to other viral proteins moreover antigens from this region, might be more exposed to be presented as capsid, is generally expressed in higher levels (Chertova et al. 2006). A further examination reported another strategy for HIV-2 targeting. Genomic RNA dimerisation is facilitated by a sequence positioned within the Psi region and that dimerisation may certainly be linked to viral packaging thus dimerisation defective viruses will be deficient in virion maturation and infectivity, possibly presenting new targets for HIV-2 replication inhibition (L'Hernault et al. 2007).

These essential demonstrations indicate cross reactivity, strong antigenicity by conserved domains especially V3, antibody response against them and generation of anti-HIV-2 V3 mouse MAbs i.e. 3C4 and 7C8. Also the difference in signal transduction in HIVs, gag specific T-cell responses and dimerisation defective HIV-2 (deficient in maturity and infectivity) give minute details about HIV-2 which can prove stepping stones while aiming it and AIDS. But these essentials alone can’t be sufficient, as some novel strategic effort which can use the above facts in a quicker way for rapid prediction of vaccine, is the need of the hour.

## Future Aspects

The search for an effective AIDS vaccine is the most daunting task because of the variable and divergent characteristic of the virus along with extremely long and time taking protocols for vaccine development which calls for a minimum duration of 6-10 years. Till today vaccine development program has reached up to phase 3 trials for HIV-1 but no validated vaccine against HIV-1/HIV-2 has been developed. The difficult and deficient character of these approaches is an obvious obstacle towards developing new vaccine.

Application of reverse vaccinology can be a possible strategy to overcome the aforementioned obstacles and surmount these challenges. It offers a promising vaccine development approach starting with the prediction of vaccine targets by bioinformatics scrutiny of pathogenic genome sequences (Rappuoli 2000). Predicted protein targets for instance, envelope protein etc. are chosen on the basis of desirable features after which normal wet laboratory trials could be carried out for testing the selected targets (Pizza et al. 2000; Rappuoli 2000) which reduces both time and cost of development. Also, other upcoming fields of vaccine research like vaccinomics including pharmacogenomics, pharmacogenetics as well as bioinformatics has proved beneficial in novel vaccine target prediction for extremely variable pathogens (Sirskyj et al. 2011). Moreover, synthetic peptides could be designed resembling the immunogenic or conserved domains of hyper variable pathogens and could be tested via the series of new emerging softwares for immune responses.

In one such study, two computer-driven methods for improving epitope-driven HIV vaccines: the *Epi-Assembler*, for deriving "immunogenic consensus sequences" (ICS) epitopes from multiple viral variants, and Vaccine CAD , which reduces junctional immunogenicity between epitopes for insertion in a DNA expression vector, were selected. They were used for selection and construction of novel “immunogenic consensus sequence” T cell epitope-driven HIV vaccines (De Groot et al. 2005). Further computational modeling was applied for recognition of chimeric HRV (Human Rhinovirus type 14) constructs. They preferentially displayed the epitope in the same β-turn conformation as already depicted in the crystal structure of complex with 2 F5 and chimeric HRV displaying the 2F5 epitope of HIV-1 gp41 (called ELDKWA after its core sequence). It signified the prospect of eliciting protective immune responses (Lapelosa et al. 2009).

Many web based servers and softwares have been developed in the recent past which can efficiently predict epitopes and consensus sequence proving to be a quantum leap in the direction of vaccine development. *MHCPEP* is a curated database comprising over 4000 peptide sequences known to bind MHC molecules. Entries are compiled from published reports as well as from direct submissions of experimental data (De Groot et al. 2002). *PEPVAC* (Promiscuous EPitope-based VACcine), optimized for the formulation of multi-epitope vaccines with broad population coverage (Reche & Reinherz 2005). *Vaxign* is the web-based vaccine design system for prediction of vaccine targets based on genome sequences using the strategy of reverse vaccinology. Predicted features comprise of protein sub cellular location, transmembrane helices, adhesion probability, conservation to human and/or mouse proteins, sequence exclusion from the genome(s) of nonpathogenic strain(s), and epitope binding to MHC class I and class II (He et al. 2010). Several other algorithms and databases for vaccine prediction are (Sirskyj et al. 2011):

- **EpiMatrix** (EpiVax Inc): Predicts epitopes for over numerous MHC class I and II alleles.
- **BlatiMer:** Automated BLAST search tool.
- **Conservatrix:** Finds conserved epitopes.
- **PAP:** Predicts protein sequences which can be antigenic by generating an antibody response
- **SYFPEITHI:** Database having numerous peptide sequences showing binding affinity with MHC class I and II molecules.

Modern strategies using *in silico* approaches will definitely be more quick, cost effective and may prove worthy in finding vaccine targets for HIV-2.

## Conclusion

HIV-2 is slow progressing, less virulent and less mortal as compared to HIV-1. But certain striking features make it a promising vaccine candidate for targeting both HIV-1 & 2 such as its cross reactivity to HIV-1, spontaneous control of HIV-1 in African monkeys (believed to be ancestors of HIV-2), existence of certain extremely conserved domains in it against which neutralizing antibody response has been observed i.e. envelope proteins as well as *gag*. Thus detailed considerations of HIV-2 can guide us towards a profound understanding of both viruses which can prove a landmark for design of a competent vaccine for AIDS

**Figure 3:**
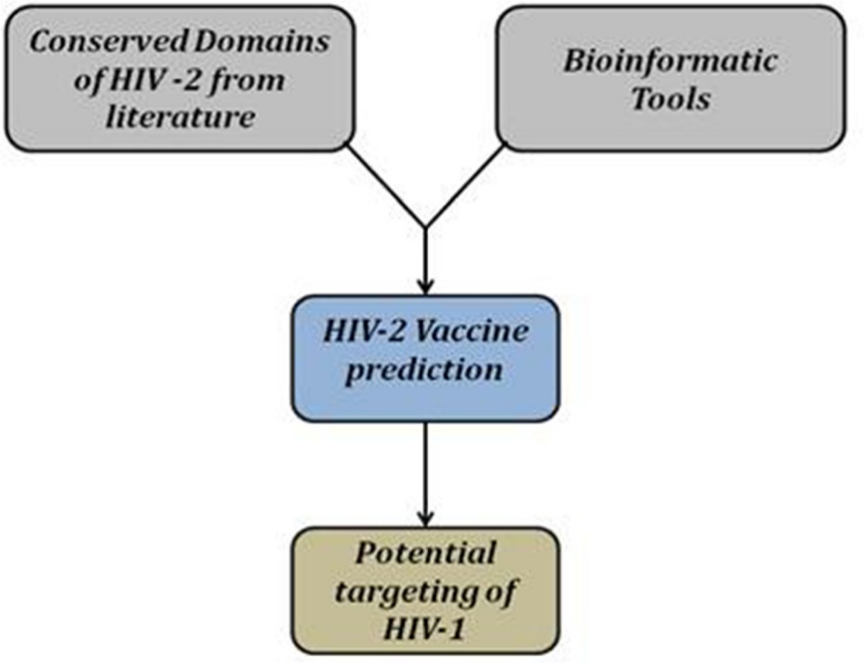
**Application of Bioinformatics tools for HIV-2 and AIDS vaccine prediction:** HIV-2 infected individuals exhibit cross protection to HIV-1 and hence HIV-2’s conserved genomic regions could be utilized for targeting HIV-1 also. Bioinformatic tools, web servers like epitope prediction tools and other softwares can ease this task and hence can make the approach novel, rapid and effective for AIDS vaccine development.

But conventional approaches are not entirely capable of dealing with various complications that arise during the development of new vaccines. Therefore the future of vaccine prediction lies in the union of current approaches like reverse vaccinology, vaccinomics by means of bioinformatics tool, web servers and softwares along with conventional approaches (Figure 3). This unification of existing conventional approaches with the above mentioned new strategies for predicting vaccine against HIV-2 will certainly be helpful in the lane of vaccine development against HIV-1 and AIDS which undeniably will be a future benediction to mankind.

## Authors’ original submitted files for images

Below are the links to the authors’ original submitted files for images. Authors’ original file for figure 1 Authors’ original file for figure 2 Authors’ original file for figure 3
