# Supplementary figures and images for: HIV-2 and its role in conglutinated approach towards Acquired Immunodeficiency Syndrome (AIDS) Vaccine Development
Source: Springerplus. 2013 Jan 11;2(1):7. doi: 10.1186/2193-1801-2-7 (PMC3586397; doi:10.1186/2193-1801-2-7)

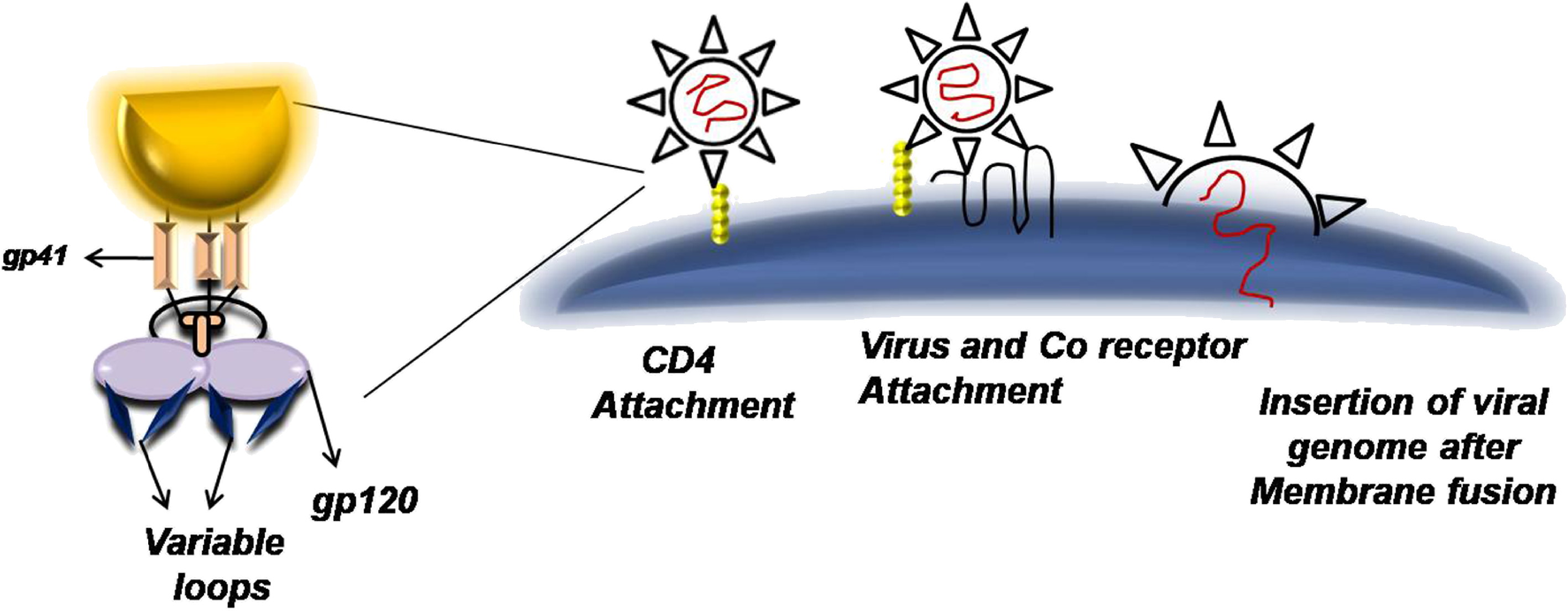

Supplement: Supplementary file 1 — Authors’ original file for figure 1 [file 40064_2012_99_MOESM1_ESM.tiff]

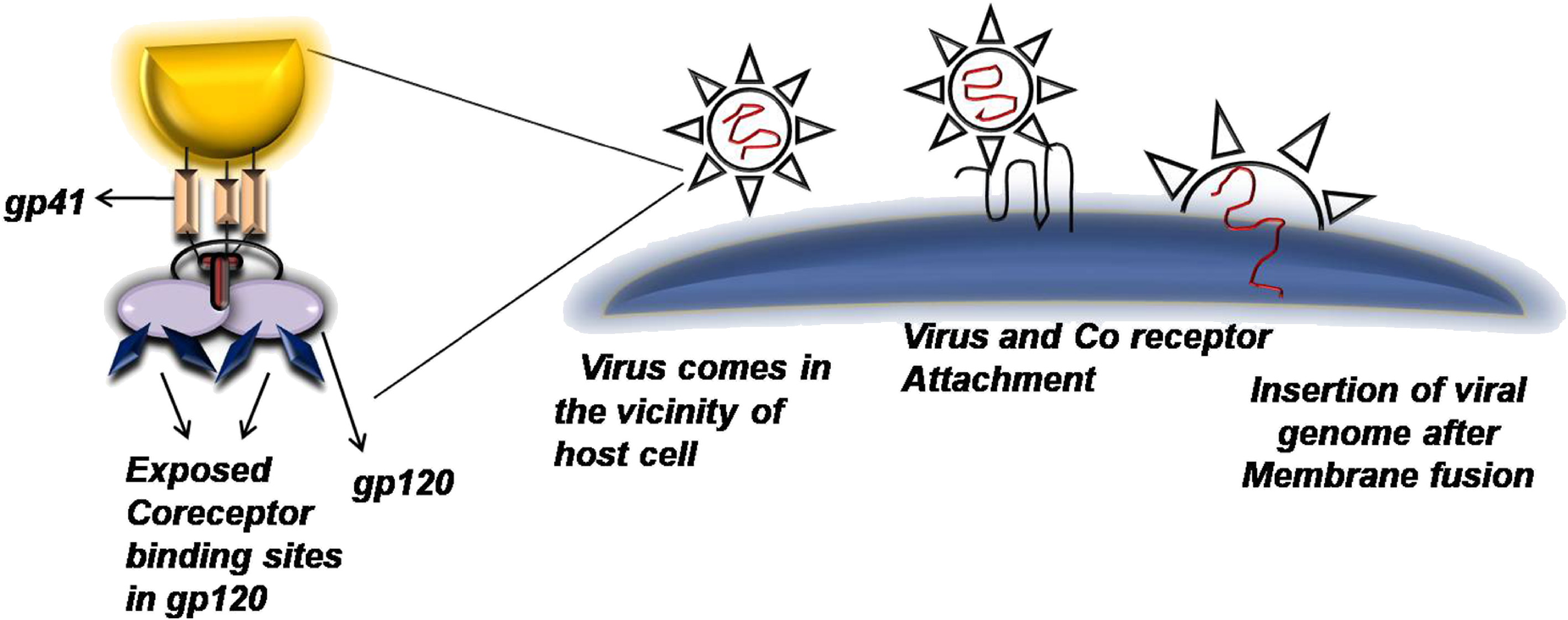

Supplement: Supplementary file 2 — Authors’ original file for figure 2 [file 40064_2012_99_MOESM2_ESM.tiff]

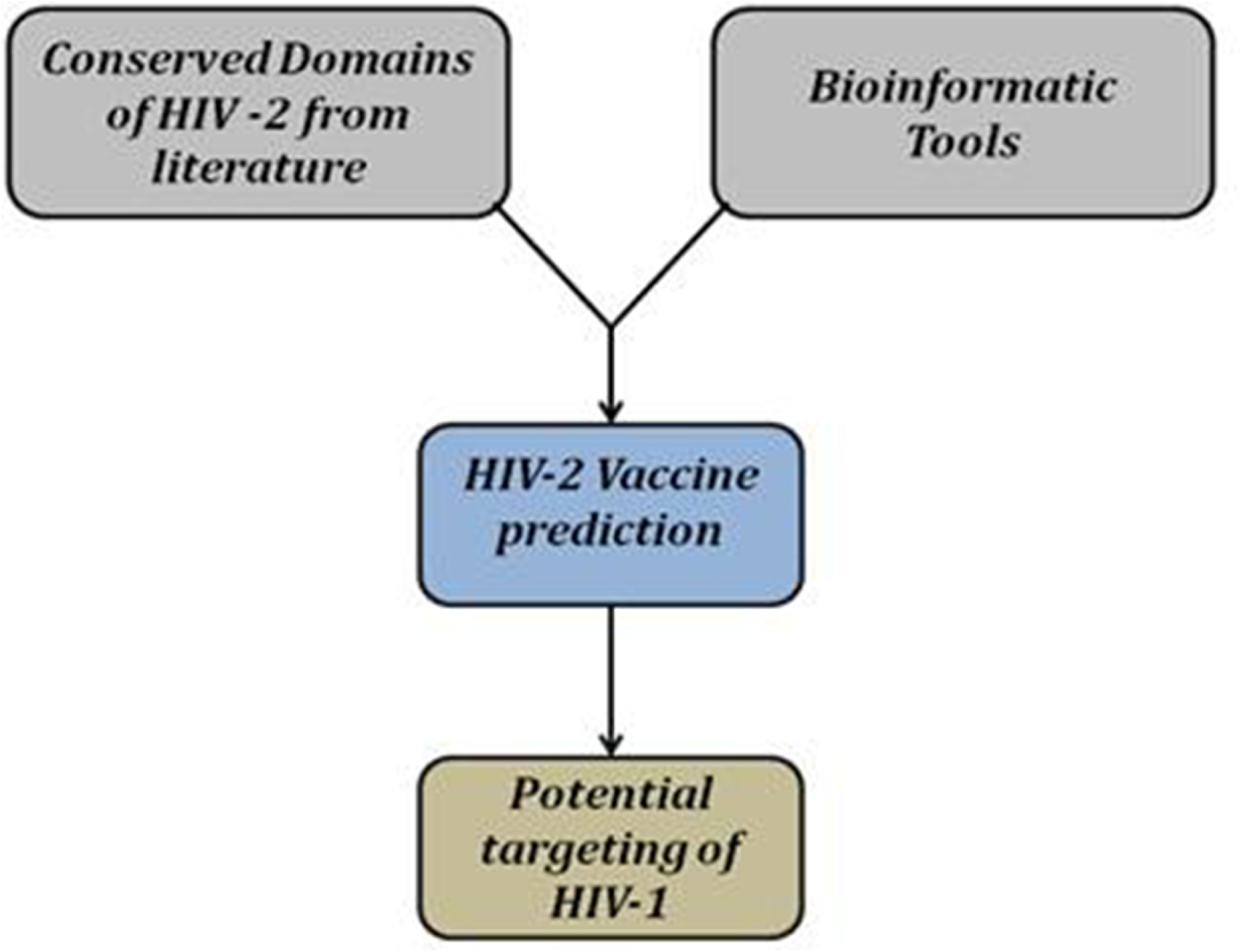

Supplement: Supplementary file 3 — Authors’ original file for figure 3 [file 40064_2012_99_MOESM3_ESM.tiff]
